# A case of nephrocalcinosis in a 7‐month‐old with congenital hypothyroidism: Insights from targeted exome sequencing

**DOI:** 10.1002/pdi3.53

**Published:** 2024-03-07

**Authors:** Omar Zgheib, Lina Quteineh, Paloma Parvex, Caterina Marconi, Valerie Schwitzgebel, Massimiliano Bertacchi

**Affiliations:** ^1^ Division of Genetic Medicine Department of Diagnostics Geneva University Hospitals Geneva Switzerland; ^2^ Division of Pediatric Nephrology Department of Pediatrics, Gynecology and Obstetrics Geneva University Hospitals Geneva Switzerland; ^3^ Division of Pediatric Endocrinology and Diabetes Department of Pediatrics, Gynecology and Obstetrics Geneva University Hospitals Geneva Switzerland

**Keywords:** congenital hypothyroidism, hyperoxaluria, nephrocalcinosis, targeted exome sequencing

## Abstract

Nephrocalcinosis is a complex disease with a multitude of triggering factors. An association with congenital hypothyroidism has been described in the literature, but the mechanisms leading to its development remain unclear. A 7‐month‐old infant presented with muscular hypotonia and signs of malnutrition was diagnosed with congenital hypothyroidism, nephrocalcinosis of unclear origin, and multiple kidney stones. Urine analysis revealed the presence of calcium oxalate crystals and slightly elevated oxaluria without hypercalciuria. Targeted exome sequencing found no variants in the four causative genes of primary oxaluria, namely *AGXT*, *CBX5*, *GRHPR*, and *HOGA1*. The clinical outcome was favorable with thyroid hormone and potassium citrate treatment. Ultrasound follow‐up showed progressive improvement of nephrocalcinosis. The low level of urinary oxalate and the prevalence of dihydrate calcium oxalate crystals spoke against primary or secondary oxaluria. Recent evidence from mitochondrial research shows that thyroid hormone T3 enhances mitochondrial calcium levels by stimulating calcium uptake by the mitochondrial calcium uniporter. A reduced calcium uptake and metabolism in mitochondria might represent an explanation for cytoplasmic calcium accumulation in tubular cells, subsequently precipitating nephrocalcinosis in the absence of thyroid hormone. Even if the pathophysiological mechanisms are not yet fully understood, recent evidence is supportive of a causal relationship between hypothyroidism and nephrocalcinosis, a sometimes overlooked association.

## 1 INTRODUCTION

The worldwide introduction of neonatal screening has improved early detection and treatment of congenital hypothyroidism, decreasing the number of cases with severe symptoms. The association of congenital hypothyroidism with other pathologies, such as nephrocalcinosis, has been episodically described but remains poorly understood.

The causes of nephrocalcinosis are multifactorial and involve the precipitation of electrolytes such as calcium, oxalate, phosphate, and uric acid in the tubules of the renal medulla. This risk of precipitation increases in concentrated urine, with elevated excretion of urine electrolytes, with change in urinary pH, or with insufficient inhibitors of crystallization like magnesium and citrate. The correlation between asymptomatic nephrocalcinosis and kidney stone risk is debated; nevertheless, nephrocalcinosis has been associated with an increased risk of developing end‐stage renal disease.

## 2 CASE REPORT

A 7‐month‐old female infant with a migratory background and no previous medical follow‐up presented with progressive asthenia and feeding difficulties, accompanied by signs of malnutrition. The initial presentation at the emergency room showed a severe dehydration state with important axial hypotonia, impossibility to maintain her head, lethargy, oliguria, an open wide anterior fontanelle, and macroglossia.

**FIGURE 1:**
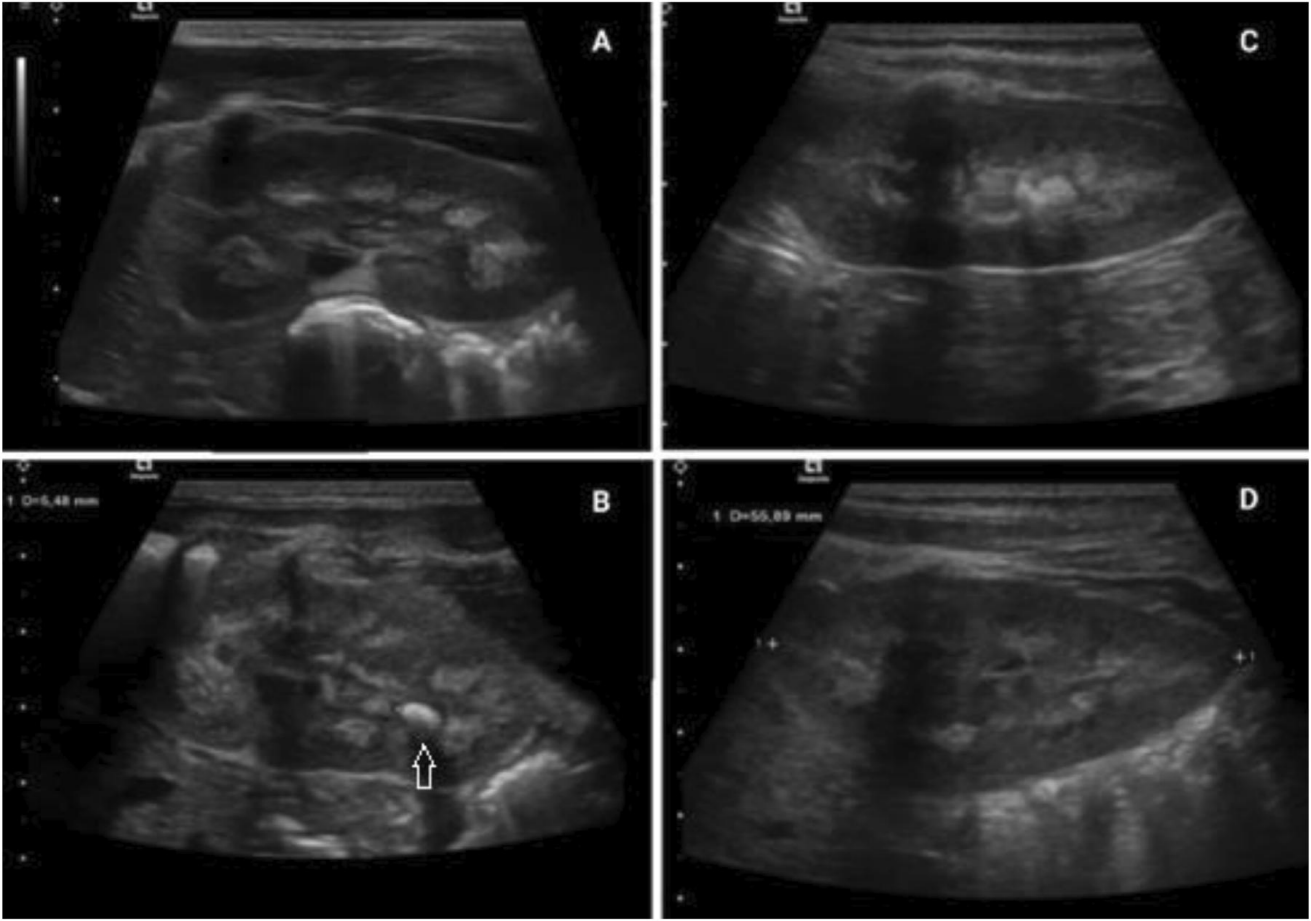
Kidney ultrasound images at the age of 8 months (A and B) showing one of three stones (arrow) and the presence of severe nephrocalcinosis and at the age of 13 months (C and D) showing improvement of medullary nephrocalcinosis 5 months after treatment with thyroid hormone and potassium citrate.

On admission, the laboratory workup showed renal failure, with creatinine at 64 umol/l and eGFR estimated at 33 ml/min/1.73 m^2^ according to the 2009 revised Schwartz equation, taking into account the patient's height (58 cm). This was resolved with rehydration, improved fluid intake, and renutrition. Nevertheless, 10 days after admission, she presented an episode of macrohematuria. Initial urine analysis showed dihydrate calcium oxalate, a small amount of monohydrate crystals, and slightly elevated oxaluria (oxalate to creatinine ratio at 585 mmol/mol, *N* < 360 under 6 months of age). The urine citrate to creatinine ratio was 0.49 mol/mol (*N* > 0.25), and the urine calcium to creatinine ratio was 0.45 mol/mol (*N* < 2.2). Calcium and phosphorus workup was normal. We measured glycolic and glyceric acid to investigate primary hyperoxaluria. The urinary glycolate to creatinine ratio was normal (114 mmol/mol, N 22‐139); glycerate was undetectable. Abdominal ultrasound showed multiple kidney stones and severe bilateral nephrocalcinosis (Figure 1A,B). Two‐week ultrasound follow‐up showed a transient 6 mm pyelocalyceal dilation, compatible with stone migration.

This child was born in Albania by C‐section after a full‐term, uneventful pregnancy. Birth parameters were normal, and neonatal adaptation was unremarkable. No neonatal screening for metabolic diseases was performed. Hypotonia and failure to thrive, manifest as weight stagnation at less than 1st percentile, were already observed at 2 months of age. Due to a complex social situation, medical follow‐up was not feasible before her arrival to Switzerland at the age of 7 months.

After an initial workup showing thyroid stimulating hormone at 449 mUI/l and undetectable T3/T4 thyroid hormone levels, the diagnosis of congenital hypothyroidism was suspected. Thyroid ultrasound showed a properly positioned homogeneous thyroid gland, with a volume in the upper normal limits for the patient's age. The above diagnosis was confirmed by pathological thyroid scintigraphy (I‐123) and a reduced perchlorate fixation test compatible with thyroid dyshormonogenesis.

Because of the clinical association of congenital hypothyroidism, anemia, macroglossia, the wide open anterior fontanelle, and nephrocalcinosis, we suspected a causative link between congenital hypothyroidism and nephrocalcinosis, but it was important to rule out a genetic etiology for the latter.

We therefore performed targeted exome sequencing using the Twist Human Core Exome capture kit (Illumina NextSeq500 sequencer) and targeted analysis of genes known to be involved in primary hyperoxaluria, namely *AGXT*, *CBX5*, *GRHPR*, and *HOGA1*. No genetic variant was found in any of these genes, but we were able to confirm the molecular basis for congenital hypothyroidism with the demonstration of two mutants in the *TPO* gene. One was a duplication of nucleotides 1184 to 1187 (NM_000547.6:c.1184_1187dup; p.Ala397Profs*76), leading to an alanine to proline mutation at position 397, followed by a frameshift, known to be a pathogenic variant. The second was a missense variant (NM_000547: c.1996G>T,p.Asp666Tyr), considered to be a likely pathogenic variant, owing to the lack of functional studies to confirm its pathogenicity. Segregation analysis confirmed the compound heterozygosity of the two variants, since only the missense variant was inherited from the mother. Paternal segregation analysis was not possible. The molecular diagnosis was of course unnecessary for the clinical diagnosis of congenital hypothyroidism but is important for genetic counseling, *TPO* deficiency being an autosomal recessive disorder.

On gradual thyroid hormone substitution, all three hormone levels normalized. The patient progressively augmented oral intake, gained weight, and improved muscular tone. As first‐line treatment for calcium oxalate stones, she received oral potassium citrate (0.5 mmol/kg/d in 2 daily doses, equivalent to 1.5 mmol/kg/d of bicarbonate). The favorable outcome was readily observed on 1‐month ultrasound follow‐up, with resolution of obstructive urolithiasis of the right kidney. Bilateral medullary nephrocalcinosis persisted, however, in addition to two stable pelvic and calyceal lithiases of the left kidney. Ultrasound follow‐up 6 months later showed improved renal parenchymal cortico‐medullary differentiation, with reduced nephrocalcinosis observed only at the base of the pyramids (Figure 1C,D) and persistence of the two left kidney lithiases.

The cause of the severe nephrocalcinosis was not clearly understood as the patient presented neither hypercalciuria nor hypercalcemia; serum calcidiol was normal, and levels of urinary oxalate were only slightly elevated with an oxalate/creatinine ratio of 217 μmol/l (*N* < 175 between 6 months and 1 year of age). Primary oxaluria was unlikely and ruled out by genetic testing. The differential diagnosis of enteric hyperoxaluria due to increased intestinal oxalate absorption secondary to enteral calcium deficiency was considered. However, the persistence of stable urinary oxalate levels after feeding normalization and the presence of a majority of dihydrate calcium oxalate crystals suggested a component of calcium dysregulation.

Evolution over the first 6 months was favorable, with clear improvement of echographic signs of nephrocalcinosis (Figure 1C,D) and the absence of macrohematuria.

The urinary oxalate to creatinine ratio increased initially to 847 mmol/mol at 1 month, possibly following the dissolution of medullary calcifications and normalized at 6 months (217 mmol/mol). Similarly, the calcium/creatinine ratio increased over time, possibly either because of a washing‐out effect of nephrocalcinosis or because of an increased alimentary calcium intake. Knee and hand radiographs showed delayed ossification as expected with hypothyroidism but normal calcium content, speaking against the hypothesis of bone degradation as the cause of nephrocalcinosis.

## 3 DISCUSSION

We suspected a causative link between congenital hypothyroidism and nephrocalcinosis, through clinical reasoning that could attribute to the former entire clinical picture (anemia, macroglossia, wide fontanelle, and nephrocalcinosis). Genetic analysis ruled out a classical form of primary hyperoxaluria, which would have required a specific therapeutic approach. The correction of hypothyroidism and a symptomatic approach with an adequate fluid intake and treatment with potassium citrate resulted in marked improvement of nephrocalcinosis.

The association of nephrocalcinosis and congenital hypothyroidism has been reported in 35 cases in the literature, the majority of which were described in a case series published in 1973.^1^ The paucity of reports is explained by the introduction of neonatal screening at the end of the 1970s, which has fortunately allowed for timely identification of newborn cases of congenital hypothyroidism, thus avoiding complications due to a lack of adequate treatment, such as nephrocalcinosis.

The pathophysiological basis of nephrocalcinosis in congenital hypothyroidism was never firmly established. It has been postulated that calcium accumulation against its concentration gradient in mitochondria of renal tubular cells could be defective in hypothyroidism, due to altered oxidative phosphorylation. This would then lead to elevated cytoplasmic calcium levels and an increased predisposition to nephrocalcinosis and nephrolithiasis.^1^ Mitochondria are present in high amounts in the renal medulla and tubular cells, and their dysfunction is associated with renal acidosis, proteinuria, and chronic kidney disease.^2^ The exact mechanism of mitochondrial calcium accumulation resulting from hypothyroidism has until recently remained unknown. Last year, Tawfik and colleagues published an original work revealing that thyroid hormone T3 enhances mitochondrial calcium levels by promoting calcium uptake by the mitochondrial calcium uniporter. This mechanism is dependent on uncoupling protein 2 and controlled by protein arginine methyltransferase 1.^3^ The reduced mitochondrial calcium uptake and metabolism in the absence of thyroid hormone provide a possible explanation for subsequently elevated cytoplasmic calcium levels, which might then amount to tubular nephrocalcinosis (Supplementary Figure S1). However, it is not entirely clear how failing to accumulate calcium in mitochondria would result in its tubular precipitation. While the former mechanism has now been identified, more research is necessary to shed light on the latter. Regardless of the mechanism, the nephrocalcinosis phenotype in this case is likely due to the hypothyroid state; there is no evidence for TPO genotype–phenotype correlation.

Interestingly, a recent report described nephrocalcinosis in a 6‐year‐old boy known for trisomy 21 and hypothyroidism. Hashimoto thyroiditis was diagnosed with positive anti‐TPO antibodies. The outcome was favorable with thyroid hormone and potassium citrate treatment, as in our patient.^4^

Other syndromes associate both hypothyroidism and nephrocalcinosis. For example, hypercalcemia (20%–40%), nephrocalcinosis (5%) and hypothyroidism (10%) are often reported in patients with Williams–Beuren syndrome (frequency indicated in parentheses).^5^ Association between hypothyroidism and hypercalcemia in these patients is well‐documented and may therefore be partly due to the pathophysiological mechanisms described above.^6^, ^7^

A report of the reverse condition is worth mentioning, whereby oxalosis caused multiorgan failure, including anemia and hypothyroidism. In this case, a 47‐year‐old patient had systemic oxalosis and was on dialysis for end‐stage renal disease. Anemia and hypothyroidism were likely a result of oxalate accumulation in the bone marrow and thyroid gland.^8^

## 4 CONCLUSION

To our best knowledge, this is the first report of targeted exome sequencing being used to rule out a genetic basis for oxaluria observed in a patient with nephrocalcinosis and congenital hypothyroidism. Our observations are supportive of a causal relationship between hypothyroidism and nephrocalcinosis, corroborated by the recently discovered mechanisms of thyroid regulation of mitochondrial calcium uptake, though more research is needed to explain tubular calcium precipitation.

Genetic exclusion of primary oxaluria was essential as the therapeutic approach would have been entirely different. The clinical outcome was favorable in our patient upon potassium citrate treatment and thyroid hormone replacement therapy. No official recommendations exist regarding the necessary duration of potassium citrate treatment; we do recommend, however, that the treatment should be pursued until resolution of nephrocalcinosis, given that it is secondary to hypothyroidism.

In light of our findings, it would be appropriate to perform renal ultrasound screening for nephrocalcinosis in those patients with late detection of congenital hypothyroidism, such as the case of our patient. This would not be necessary in neonatally diagnosed congenital hypothyroidism. It is arguable to extend this screening to patients known for Williams–Beuren syndrome or trisomy 21 and presented with hypothyroidism.

## AUTHOR CONTRIBUTIONS

OZ and MB wrote the manuscript. CM contributed to molecular analysis of hypothyroidism and hyperoxaluria genes. OZ, LQ, MB, PP, and VS contributed to the clinical diagnosis and follow‐up. All provided critical revision of the manuscript.

## CONFLICT OF INTEREST STATEMENT

The authors declare no conflicts of interest.

## ETHICS STATEMENT

Written informed consent for publication of their clinical details and clinical images was obtained from the mother. A copy of the consent form is available for review by the editor on request.

## Supporting information

Figure S1

## Data Availability

The data that support the findings of this study are available from the corresponding author upon reasonable request.
