# Supplementary material for: A case of nephrocalcinosis in a 7‐month‐old with congenital hypothyroidism: Insights from targeted exome sequencing
Source: Pediatr Discov. 2024 Mar 7;2(1):e53. doi: 10.1002/pdi3.53 (PMC12118225; doi:10.1002/pdi3.53)
Supplement: Supplementary file 1 — Figure S1 [file PDI3-2-e53-s001.docx]

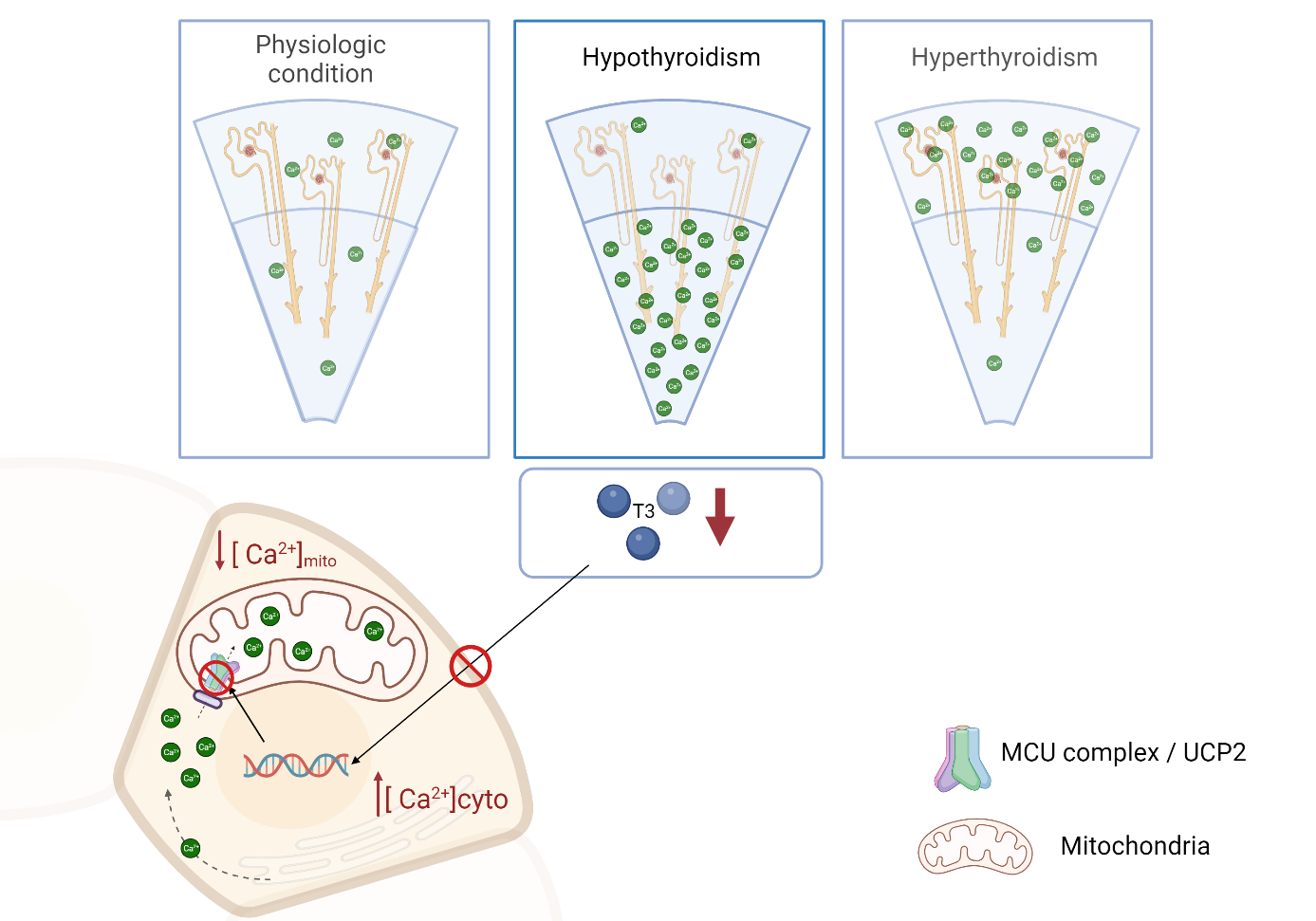
 **Supplementary Figure.** Pathophysiological model of cytosolic calcium accumulation in the absence of thyroid hormone and schematic of calcium kidney medullary distribution in physiologic, hypothyroid and hyperthyroid states. Adapted from I. Tawfik (*Free Radic. Biol. Med.,* 2022) and R. J. Newman (*J. Pathol.,* 1973).
